# Low Femoral Tunnel Widening Incidence Rate After ACL Reconstruction Using Patellar Tendon Graft with Press-Fit Fixation

**DOI:** 10.1007/s43465-023-00836-3

**Published:** 2023-02-24

**Authors:** Miklós Tátrai, Tamás Halasi, András Tállay, Annamária Tátrai, Attila Pavlik

**Affiliations:** 1 Department of Orthopedic Surgery, Kastélypark Clinic, Hajdú Street 15., Tata, 2890 Hungary; 2 Faculty of Sport Medicine, Semmelweis University, Gaál József Street 9-11., Budapest, 1122 Hungary; 3 Faculty of Social Sciences, Eötvös Lóránd University, Pázmány Péter Promenade 1/A, Budapest, 1117 Hungary; 4 Budapest, Hungary

**Keywords:** Femoral tunnel widening, Patellar tendon, Press-fit fixation, ACL reconstruction

## Abstract

**Background:**

Femoral tunnel widening after ACL reconstruction is a common phenomenon. We hypothesized that using a patellar tendon graft with a press-fit fixation technique without any fixation device reduces the incidence of femoral tunnel widening.

**Methods:**

This study was conducted on 467 patients with ACL surgery between 2003 and 2015. Two hundred and nineteen of them had an ACL surgery with patellar tendon (PT) graft, and two hundred and forty-eight of them with hamstring tendon (HS). Exclusion criteria were history of previous ACL reconstruction of either knee, multiple ligament injury, or evidence of osteoarthritis on radiographs. The femoral tunnels were measured on the anteroposterior (ap) and lateral radiographs 6 months after the operation. Two independent orthopedic surgeons measured all radiographs twice and recorded the tunnel widenings. We hypothesized that using an implant-free press-fit technique with PT graft can reduce the femoral tunnel widening incidence rate.

**Results:**

The mean incidence rate of the tunnel widening in the HS group was, on the AP and the lateral femoral views, 88% (*n* = 217) and 83% (*n* = 205), while in the PT group, 17% (*n* = 37) and 2% (*n* = 4), respectively. There was a significant difference both on AP and lateral radiographs (HS vs. PT: fem. AP: 89% vs. 17% *p* < 0.001; HS vs. PT: fem. lat: 84% vs. 2% *p* < 0.001).

**Conclusion:**

The femoral tunnel widening incidence rate during an ACL reconstruction is significantly less when using PT tendon with femoral press-fit fixation than when using HT tendon with suspensory fixation method.

## Introduction

The tunnel widening after anterior cruciate ligament (ACL) reconstruction is a common, well-known phenomenon. Its incidence ranges from 0 to 74% [1]. The widening is more marked with the use of hamstring (HS) graft than with the use of patellar tendon (PT) graft [2–5]. Besides the graft type, a number of factors can trigger it, such as movement of the graft within the tunnel, the age of the patient, accelerated rehabilitation, the size and the position of the drilled holes, the different types and devices of the graft fixation, and higher cytokine activity [6–13]. The exact etiology of tunnel widening is still unknown. A common theory is that synovial fluid inflow occurs inside the tunnel between the graft and the bone, leading to a series of disorders in the normal bone–tendon healing process [13]. After the surgery, there is an improvement in the proinflammatory cytokine (TNF-a, IL-1b, IL-6) levels in the intra-articular fluid [12, 15]. These cytokines stimulate osteoclast activity, contributing to bone resorption [16, 17]. Due to the movement of the graft, the synovial fluid can leak into the tunnels [1]. As confirmed by observation, accelerated rehabilitation leads to greater tunnel widening [10]. It may increase or prolong the exposure of the adjacent bone to the synovial fluid if the graft is relatively flat in a round bone tunnel. This is referred to as the “synovial bathing effect” [12, 14]. If we reduce graft movement in the bone tunnel (windshield-wiper effect) with a fixation method (e.g., interference screw, press-fit technique) next to the joint line, we can decrease the synovial inflow. However, the most commonly used cannulated interference screw has a central hole as well as a space between the screw thread and the bone, both of which can cause synovial inflow. If we use the press-fit fixation technique, we can close the aperture of the femoral tunnel with the base of the bone block.

Nevertheless, no study in the literature evaluates the effect of the ACL reconstruction with the patellar tendon press-fit technique and the incidence rate of femoral tunnel widening.

The purpose of this study was to compare femoral bone tunnel widening after ACL reconstruction with suspensory HS and with press-fit PT graft.

We hypothesized that the implant free press-fit technique with PT graft would lead to less femoral tunnel widening.

## Methods

### Patients

This retrospective study was conducted on 467 patients with ACL surgery in our sports surgery department between 2003 and 2015. Exclusion criteria were (1) history of previous ACL reconstruction of either knee, (2) multiple ligament injury, (3) evidence of osteoarthritis on radiographs. The concomitant meniscal injury was not an exclusion criterion. Two groups were created according to the graft used, the hamstring (HS) group and the patellar tendon (PT) group (HS group: *n* = 248; PT group: *n* = 219). Patient data are summarized in Table 1. There was no significant difference between the average age of the groups. However, the operation time was significantly longer in the PT group than in the ST group (HS:44 min; PT 53: min; *p* < 0.001; 95%CI − 11.53 to − 7.02). In both groups, there were more males than females, and the difference between the groups was significant (HS: M:F 168:80; PT: M:F 180:39; *p* = 0.0005).

**Table 1:** Comparison to groups

	Hamstring (HS *n* = 247)	Patellar tendon (PT *n* = 219)	*p*	95% CI
Age	27.4 years (13.5–57.2)	27,9 years (14.5–51.7)	0.528	− 378.62 to 737.18
Sex	M:F 168:80	M:F 180:39	0.0005	n.a
Operation time (min)	44 min (25–100)	53 min. (30–100)	0.001	− 11.53 to − 7.02

### Operation Techniques

The operations were performed in the HS group with quadrupled semitendinosus and gracilis tendon graft using a suspensory fixation system with endobutton at the femoral and two spiked staples at the tibial end. The single-bundle reconstruction was precisely described by Kawaguchi et al. [18].

The bone–patellar tendon–bone graft was harvested with the technique in Pavlik et al. [19] The most important step of this technique was to very accurately shape the patellar bone. It had a trapezoid form with most commonly 9 mm diameter at the end and 10 mm diameter at the base. In this case, the patellar bone block was impacted in the 9 mm wide femoral tunnel. The femoral tunnel and the patellar bone had the same length; hence the base of the patellar bone fit totally into the femoral tunnel, without any fixation device. The same technique was used with the trapezoid form at the tibial tunnel.

All of the operations were performed by one senior surgeon (TH). All procedures performed in studies involving human participants were in accordance with the ethical standards of the institutional and/or national research committee and with the 1964 Helsinki Declaration and its later amendments or comparable ethical standards. The study was approved by the regional ethical committee. (No. 16/2019).

### Rehabilitation

The patients had the same rehabilitation protocol. After the surgery, they were wearing a 0° fixed brace for 3 weeks. The patients practiced isometric muscle stretching and 30°–40° flexion during this time. We allowed full weight-bearing 1 week after the operation. On the fourth postoperative week, full range of movement was initiated. Bicycling was allowed after 3 weeks, swimming was allowed after 10 weeks, and straight-line running was allowed after 12 weeks. Sport-specific exercises were started on week 16. We allowed the return to sport at the ninth postoperative month.

We used the same procedure and rehabilitation program in both groups.

### Radiological Assessment

**Fig. 1:**
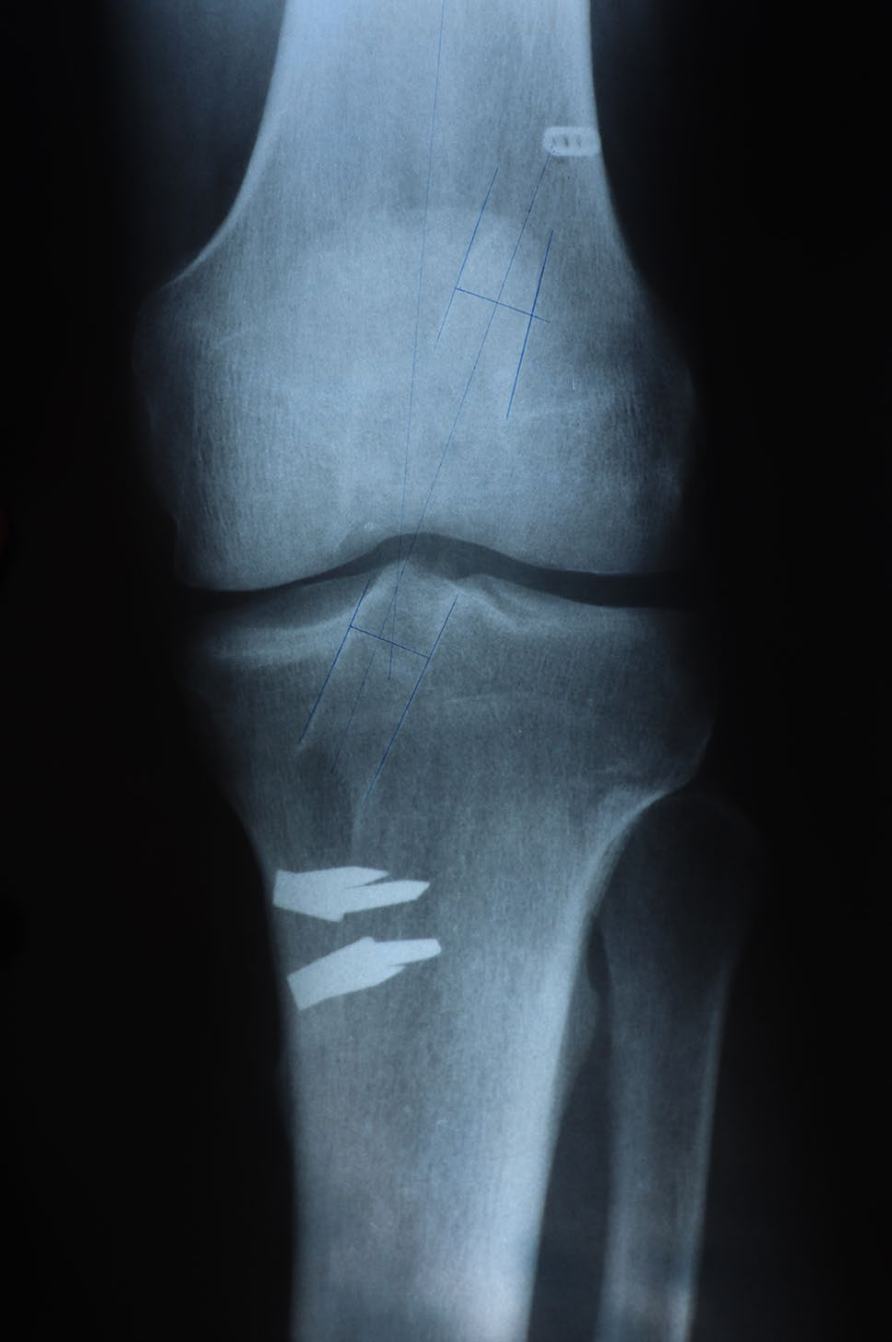
Tunnel widening on the AP X-ray, using HT graft

**Fig. 2:**
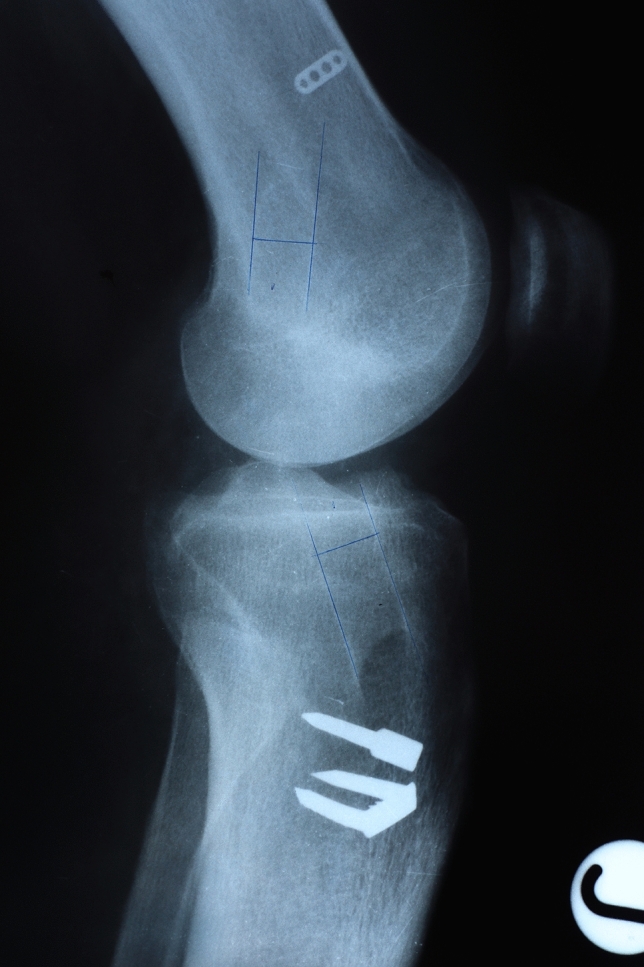
Tunnel widening on the lateral X-ray, using HT graft

**Fig. 3:**
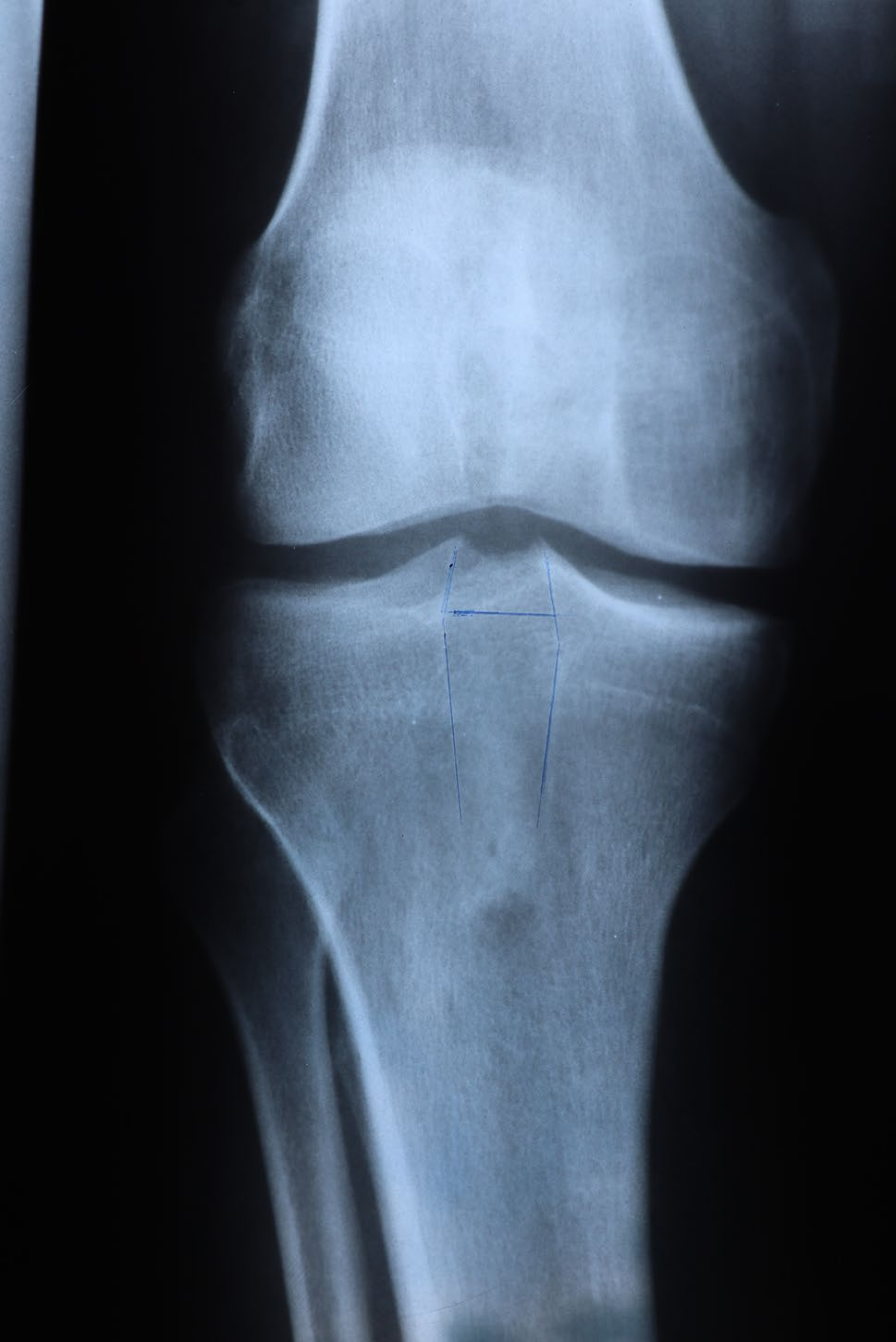
Tunnel widening on the AP X-ray, using PT graft

**Fig. 4:**
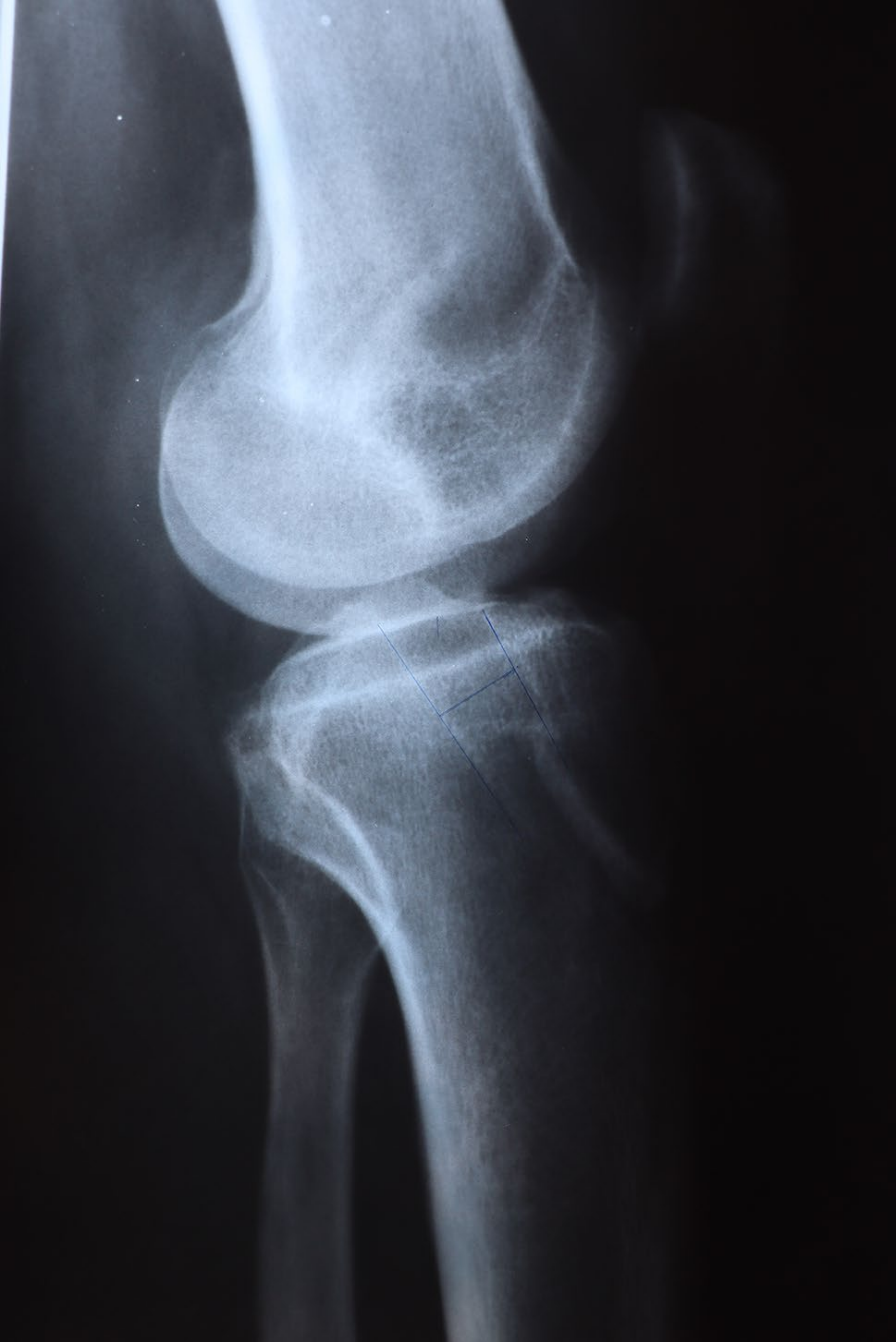
Tunnel widening on the lateral X-ray, using PT graft

Standard anteroposterior (AP) and lateral view X-rays were performed at 6 months postoperatively. The femoral and tibial tunnel widenings were measured 1 cm far from the aperture of the tunnels, perpendicular to the axis of the tunnels. We defined it as the distances between the two sclerotic bone margins. The comparison was made between the measured value and the drilled tunnels. Figures 1, 2, 3, 4.

### Statistical Analysis

Comparisons between groups with regard to age and operation time were performed using two-sample *t* tests. The ratio of the tunnel widenings was compared using a one-sample *t* test. The difference in the tunnel widenings between the groups was compared using a two-sample *t* test. We used the free R software for the statistical analysis. (R version 3.6.3. The R Foundation for Statistical Computing). The significance level was set at *p* = 0.05.

### Ethical Approval

Ethical approval for this study was obtained from the Scientific and Ethical board of the National Institute for Sports Medicine, Budapest, Hungary.

## Results

### Tunnel Widening in the Groups

The mean intraoperative tunnel diameter was at the femoral and tibial ends 7.4 mm (6–10 mm) in the HS group and 9.98 mm (9–12 mm) at the femoral, and 8.9 mm (8–10 mm) at the tibial end in the PT group. The mean incidence rate of tunnel widening was, in the HS group, on the AP and the lateral views, 88% (*n* = 217) and 83% (*n* = 205) at the femoral end, and 97% (*n* = 240) and 97% (*n* = 240) at the tibial end. The incidence rate of tunnel widening, in the PT group was 17% (*n* = 37) and 2% (*n* = 4) on the femoral AP and lateral views, and 94% (*n* = 205) and 84% (*n* = 184) on the tibial views.

There was a significant difference in the incidence rate of femoral tunnel widenings both on AP and lateral radiographs (HS vs. PT: fem. AP: 89% vs. 17% *p* < 0.001; HS vs. PT: fem. lat: 84% vs. 2% *p* < 0.001) (Table 2).

**Table 2:** Mean incidence rates of tunnel widening

	HS (*n* = 248)	PT (*n* = 219)	*p*
Femoral AP	88% (*n* = 217)	17% (*n* = 37)	0.001
Femoral lateral	83% (*n* = 205)	2% (*n* = 4)	0.001
Tibial AP	97% (*n* = 240)	94% (*n* = 205)	0.163
Tibial lateral	97% (*n* = 240)	84% (*n* = 184)	0.001

On the tibial radiographs, a higher widening rate could be observed in the HS group, although the difference was significant only on the lateral images. (HS vs. PT: tib. AP: 97% vs. 94% *p* = 0.163; HS vs. PT tib. lat: 97% vs. 84% *p* < 0.001).

The mean tunnel widening in the HS group was, on the femoral AP and lateral views, 4.15 mm (50%) and 4.18 mm (47%), and 2.86 mm (38%) and 3,0.4 mm (41%) on the tibial views. The mean tunnel widening in the PT group was 3.48 mm (6%) and 2.2 mm (0.5%) on the femoral AP and the lateral views, and 3.8 mm (36%) and 3.19 mm (28%) on the tibial views. Except for the lateral femoral radiograph in the PT group, all of the tunnel widenings were significant. (*p* < 0.001).

Because the incidence rate was low in the PT group on the femoral lateral X-ray, the statistical analysis was not possible in all cases. With this in mind, the tunnel widening on all views was significantly less in the PT group, except for the above-mentioned radiograph (fem. AP: 50–6% *p* < 0.001 95% CI 0.40—inf; fem. lat: 47–0.5% *p* < 0.001 95% CI 0,44—inf.; tib. AP 38–36% *p* = 0.9 0.05—inf; tib. lat: 41–28% *p* < 0.001 95% CI 0.09–0.17).

## Discussion

The most important finding of this study was the significantly lower femoral tunnel widening incidence rate following ACL reconstruction using press-fit fixed patellar tendon graft compared to using the suspensory-fixed semitendinosus graft. This is the first study to examine the effect of ACL reconstruction with press-fit fixed patellar tendon technique on femoral tunnel widening.

Except for the tibial AP view, we found significantly higher tunnel widening incidence rates in the HS group on all of the radiographs (HS vs. PT: fem. AP: 89% vs. 17% *p* < 0.001; HS vs. PT: fem. lat: 84% vs. 2% *p* < 0.001, HS vs. PT: tib. AP: 97% vs. 94% *p* = 0.163; HS vs. PT tib. lat: 97% vs. 84% *p* < 0.001). The incidence rates of the tunnel widening show relatively high variety (between 0 and 90%) [1]. Most authors agree that this phenomenon mostly occurs when using a hamstring graft. Hersekli found the rate of femoral tunnel widening to be more than twice as high using HS graft (100%) than PT graft (46%). The difference was shown at the tibial end as well, but that difference was not statistically significant (HS:PT 82%:76%) [4]. In our study, the tibial widening incidence rate was also higher than the femoral incidence rate in both groups, although the femoral rates were much lower than in the referenced studies in the PT group (fem. AP: 17%, fem. lat: 2% vs. 46%, 76%).

The tunnel widening after ACL reconstruction is more often seen if hamstring graft is used, compared to patellar tendon grafts, and the widening is more pronounced in the femoral end than in the tibial [1–5, 20]. Except for the femoral tunnel widening on the lateral view in the PT group, all of our tunnel widening rates were significant in both groups, and the incidence rates were the highest in the HS group (HS: fem. AP:50% *p* < 0.001, fem. lat: 47% *p* < 0.001, tib. AP: 38% *p* < 0.001, tib. lat: 41% *p* < 0.001; PT: fem AP: 6% *p* < 0.001, fem. lat: 0.5% *p* = 0.02, tib. AP: 36% *p* < 0.001, tib. lat: 28% *p* < 0.001). The difference in the widening rates was observed only on the tibial AP views and was not significant between the two groups.

Several theories exist on which factors play a role in tunnel widenings, such as the different types of grafts, the movement of the graft within the tunnel, the age of the patient, accelerated rehabilitation, the size and the position of the drilled holes, the different types and devices of the graft fixation, and higher cytokine activity [6–13]. Our theory is that during knee movement, the graft is stretched to the wall of the tunnel, it is compressed to the bone, and as a result, a space is created between the graft and the bone where the synovial fluid can leak into the tunnel [1]. The synovial fluid contains a large number of proinflammatory cytokines (TNF-a, IL-1b, IL-6) which increase osteoclast activity, leading to bone resorption, and thus tunnel widening [14, 16, 17]. We hypothesized that if the aperture of the femoral tunnel is closed with a bone block without any fixation devices and if we prevent the synovial inflow to the tunnel, it can cause less femoral tunnel widening. This theory can be supported by the study of Hollis et al., who placed autologous bone plugs in the femoral tunnel aperture, and they found less femoral tunnel widening, although the difference was not significant [21]. In case the tibial bone block was flipped next to the patellar tendon, it was closer to the joint line, decreased the synovial fluid inflow, and reduced the tibial tunnel widening significantly [16]. Other authors found that the use of the graft fixation method close to the joint line can reduce the movement of the graft within the tunnel, thereby it leads to less tunnel widening [14]. Fauno et al. achieved significantly less femoral and tibial tunnel widening using the transfix system with femoral and tibial PLLA interference screws compared to the endobutton suspensory fixation technique [7]. Conversely, Buelow et al. reported higher femoral tunnel widening with PLA interference screw fixation than with endobutton using a hamstring graft [6]. In our cases, the fixation of the femoral bone block was close to the joint line. We hypothesize that both factors lead to low tunnel widening rates and less femoral tunnel widenings.

The aim of an ACL reconstruction is to make the knee joint stable. The graft, the fixation method, the operation technique have an important role in the success of the surgery. Using PT graft is a common technique; it has the same result as using HT or quadriceps tendon graft [22]. There are a lot of possibilities for femoral graft fixation, such as interference screws, transfix method, suspensory systems, or the press-fit technique. The most important questions are: Does the femoral press-fit fixation method provide good graft stabilization, and can it offer good long-term result? Pavlik et al. measured satisfying failure strength using the femoral press-fit fixation at a pig ACL reconstruction [23]. Arnold et al. found the same primary stability with ultimate load to failure pulls forces at least equal to published results for interference screws [24]. Hertel et al. found excellent clinical results 10 years after ACL reconstruction using PT graft with press-fit fixation method [25]. Widuchowski et al. published the same good results at 15-year follow-up after ACL surgery using the femoral press-fit technique [26]. Sarzaeem found comparable results between using the press-fit and interference screw fixation [27]. Using the press-fit fixation technique yields low graft failure and revision rates [28].

This study has several limitations. First, the design of the study is just a retrospective analysis. Therefore, the enrollment of the patients was not randomized. There was a change in the operating technique in 2008, when the surgeon changed the type of graft from the patellar tendon to the hamstring tendon. We analyzed both groups, but by doing so, we could not ensure randomized patient enrollment. Second, we measured the tunnel widening on the X-ray images, rather than on CT or MRI scans. The divergence of the X-ray beams cause a magnification effect, which depends on the distance between the X-ray machine and the joint and the distance between the joint and the X-ray film cassette [14]. We used standard properties for all radiographs. Therefore, the tendency is similar on the images. Nevertheless, our most important finding is the significantly less femoral tunnel widening incidence rate when using PT graft (PT: AP 17%, lat: 2% vs. HT: AP 88%, lat: 83%), and it is independent of the magnification effect.

### Recommendation

In summary, performing an ACL reconstruction with an implant-free technique can reduce the cost of the surgery. In the absence of fixation devices, a revision ACL operation is much easier, as neither implant removal nor bone marrow filling is necessary.

## Conclusion

The femoral tunnel widening incidence rate during an ACL reconstruction is significantly lower when using PT tendon with femoral press-fit fixation than when using HT tendon with suspensory fixation method.
